# A Theoretical Study of the N to O Linkage Photoisomerization Efficiency in a Series of Ruthenium Mononitrosyl Complexes

**DOI:** 10.3390/molecules22101667

**Published:** 2017-10-06

**Authors:** Juan Sanz García, Francesco Talotta, Fabienne Alary, Isabelle M. Dixon, Jean-Louis Heully, Martial Boggio-Pasqua

**Affiliations:** 1 Laboratoire de Chimie et Physique Quantiques, IRSAMC, CNRS et Université Toulouse 3, 118 route de Narbonne, 31062 Toulouse, France; talotta@irsamc.ups-tlse.fr (F.T.); fabienne.alary@irsamc.ups-tlse.fr (F.A.); dixon@irsamc.ups-tlse.fr (I.M.D.); j-l.heully@irsamc.ups-tlse.fr (J.-L.H.); 2 Present address: Institut de Recherche de Chimie Paris, PSL Research University, CNRS, Chimie ParisTech, 11 Rue Pierre et Marie Curie, F-75005 Paris, France

**Keywords:** photoisomerization, photochromism, computational photochemistry, density functional theory

## Abstract

Ruthenium nitrosyl complexes are fascinating versatile photoactive molecules that can either undergo NO linkage photoisomerization or NO photorelease. The photochromic response of three ruthenium mononitrosyl complexes, *trans*-[RuCl(NO)(py)_4_]^2+^, *trans*-[RuBr(NO)(py)_4_]^2+^, and *trans*-(Cl,Cl)[RuCl_2_(NO)(tpy)]^+^, has been investigated using density functional theory and time-dependent density functional theory. The N to O photoisomerization pathways and absorption properties of the various stable and metastable species have been computed, providing a simple rationalization of the photoconversion trend in this series of complexes. The dramatic decrease of the N to O photoisomerization efficiency going from the first to the last complex is mainly attributed to an increase of the photoproduct absorption at the irradiation wavelength, rather than a change in the photoisomerization pathways.

## 1. Introduction

Photochromic systems are made of photoswitchable molecular building blocks, which often undergo a photoisomerization process [1,2,3]. While largely dominated by photoinduced electrocyclic reactions of organic compounds [4,5,6,7,8,9], photochromic systems have also been designed with metal complexes capable of undergoing linkage photoisomerization of ambidentate ligands (e.g., NO, RR’SO, and SO_2_) coordinated to a metal center [10,11,12,13,14]. In such inorganic systems, the photochemical and photophysical properties can be tuned by varying the nature of the different ligands. Photochromic molecular systems have entered the new generation of innovative functional materials with high added value. Applications are already widespread in nanosciences, biology, and photonic or optoelectronic devices as light-activated switches [15,16,17,18,19,20,21].

While many organic photochromic systems have been scrutinized from an experimental point of view, sometimes complemented by theoretical studies [22,23,24,25,26,27,28,29,30,31,32,33], studies of photochromic inorganic systems are much scarcer [10,11,12,13,14]. Owing to the difficulty of computing photochemical pathways in metal complexes [34], the number of theoretical studies devoted to the linkage photoisomerization mechanisms in these systems is unsurprisingly low [35,36,37,38,39,40,41,42]. Among these systems, ruthenium nitrosyl complexes have attracted considerable interest due to both their photochromic properties [43,44,45,46,47,48,49,50,51,52,53,54,55] and their capability to release nitric oxide [56,57,58,59,60,61,62,63,64,65,66]. Recently, the N→O linkage photoisomerization mechanism in the *trans*-[RuCl(NO)(py)_4_]^2+^ (where py denotes a pyridine ligand) ruthenium nitrosyl complex was investigated based on density functional theory (DFT) calculations of the lowest singlet and triplet potential energy profiles along the N→O linkage photoisomerization coordinate [41,42]. These studies brought some important insight into the mechanistic picture of the N→O linkage photoisomerization. In contrast to other photoisomerizable ruthenium complexes, for which energetically favorable routes on the lowest triplet-excited state were characterized [36,37,38], the triplet pathway for the N→O linkage photoisomerization reveals an activated and energetically uphill process, which forbids an adiabatic mechanism in this electronic excited state [41,42]. Thus, a complex sequential two-photon photoisomerization mechanism involving nonadiabatic processes was proposed [41] and confirmed experimentally very recently [54]. This mechanistic picture is supported by accurate multiconfigurational wavefunction-based calculations [67].

While a single crystal of *trans*-[RuCl(NO)(py)_4_](PF_6_)_2_·1/2H_2_O presents a quasi-complete photoconversion yield between the stable N-bonded nitrosyl (denoted **GS**) and metastable O-bonded isonitrosyl (denoted **MS1**) isomers under continuous irradiation in the 473–476 nm region [51,52,53], much lower efficiencies were observed for *trans*-[RuBr(NO)(py)_4_](PF_6_)_2_ and *trans*-(Cl,Cl)[RuCl_2_(NO)(tpy)](PF_6_) (where tpy denotes a terpyridine ligand) with yields of 46% and 8%, respectively, under comparable blue light irradiation [53]. This drastic decrease of photoconversion yield may be resulting from two main factors: (i) modifications in the potential energy surfaces (PES); and/or (ii) changes in the optical properties of the isomers involved. In the first case, the relative stabilities of the **GS** and **MS1** isomers on the ground-state PES is a critical feature, but, also, the nonadiabatic photoisomerization pathway could be modified in a way that the photoconversion becomes less efficient. Regarding the optical properties, they could be modified in such a way that either the system does not absorb light so efficiently or the photoproduct (**MS1**) also absorbs in the same spectral region, inducing the backward photoisomerization.

**Figure 1:**
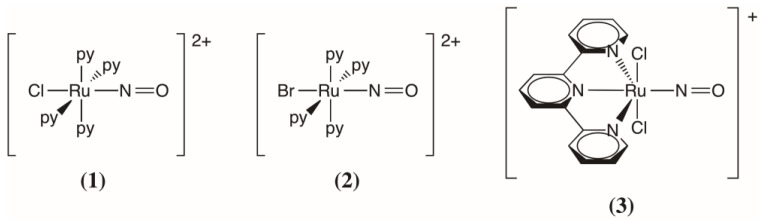
The series of ruthenium nitrosyl complexes studied in this work. From left to right: *trans*-[RuCl(NO)(py)_4_]^2+^ **(1)**; *trans*-[RuBr(NO)(py)_4_]^2+^ **(2)**; and *trans*-(Cl,Cl)[RuCl_2_(NO)(tpy)]^+^ **(3)** (py: pyridine, tpy: terpyridine).

The aim of this theoretical study is to shed light on these different aspects in order to rationalize qualitatively the trend in the photoconversion yield in this series of ruthenium nitrosyl complexes, shown in Figure 1. DFT calculations were thus performed on the isolated *trans*-[RuBr(NO)(py)_4_]^2+^ complex (denoted complex **(2)** hereafter) to determine and compare the potential energy landscape of the lowest singlet and triplet states with those of the *trans*-[RuCl(NO)(py)_4_]^2+^ (complex **(1)**) and *trans*-(Cl,Cl)[RuCl_2_(NO)(tpy)]^+^ (complex **(3)**) complexes previously studied [41,42,67]. Time-dependent DFT (TD-DFT) calculations were performed to determine the optical properties of all the relevant stable and metastable isomers involved in the photoisomerization mechanism. In the following, we present the results of these calculations and show that the main reason for the decrease of photoconversion efficiency in this series of complexes involves an increase of the photoproduct absorption at the irradiation wavelength rather than a change in the photoisomerization pathways.

## 2. Results

### 2.1. Overview on the Mechanistic Picture for the trans-[RuCl(NO)(py)_4_]^2+^ Complex

**Figure 2:**
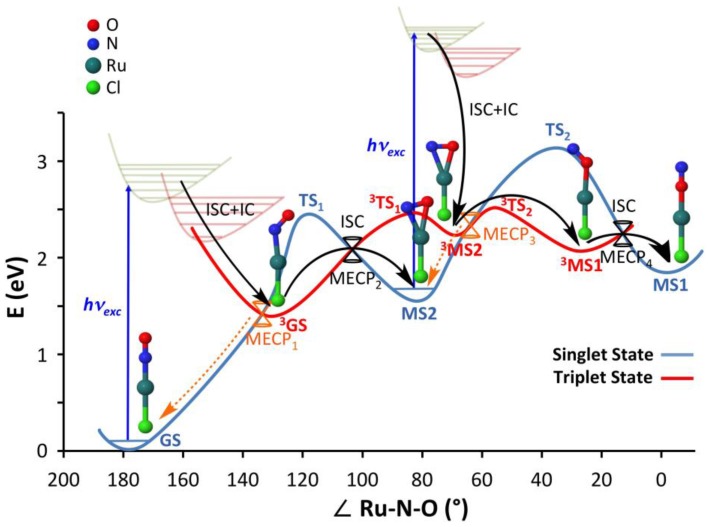
Potential energy profiles for the singlet ground state (blue curve) and the lowest triplet state (red curve) along the N→O linkage photoisomerization pathway derived from density functional theory (DFT) calculations of complex (**1**) [41]. The reaction coordinate is represented as the Ru-N-O angle. **GS**, **MS2,** and **MS1** correspond to the N-bonded, sideways NO-bonded, and O-bonded isomers in the singlet state, respectively. **^3^GS**, **^3^MS2,** and **^3^MS1** correspond to the N-bonded, sideways NO-bonded, and O-bonded isomers in the triplet state, respectively. **TS** and **^3^TS** denote transition states lying on the singlet and triplet potential energy surfaces (PES), respectively. ISC and IC stand for intersystem crossing and internal conversion. Double-cone pictograms represent the minimum energy crossing points (MECP) between the singlet and triplet states (in black are the two ones relevant to N→O linkage photoisomerization). The black arrows indicate the sequential steps of the N→O linkage photoisomerization mechanism. The orange dashed arrows and orange double cones represent photostabilizing non-radiative decay paths to **GS** and **MS2**. Pyridine ligands have been omitted in the structures for clarity.

Figure 2 summarizes the mechanistic picture that was derived from a previous DFT study of complex (**1**) [41]. Upon blue light irradiation, the most stable N-bonded ground-state isomer **GS**, in which the Cl, Ru, N, and O atoms are collinear, is promoted to excited singlet metal-to-ligand charge transfer (^1^MLCT) states. These states are known to quickly relax by intersystem crossing (ISC) due to significant spin-orbit coupling (SOC) and by internal conversion (IC) to lower triplet MLCT excited states (^3^MLCT) [67,68]. Assuming that the lowest ^3^MLCT is mainly populated, the system then relaxes to a N-bonded triplet state (denoted **^3^GS**) in which the Ru–N–O is bent. A large barrier (ca. 1 eV) on the adiabatic triplet PES forbids the system to reach a sideways NO-bonded intermediate (denoted **^3^MS2**). However, a more accessible route is provided by ISC at a triplet/singlet crossing point, allowing the system to decay at a sideways NO-bonded intermediate on the singlet state (denoted **MS2) [67]**. A key feature of this mechanism is that the **MS2** metastable state can also absorb the same blue light photon as **GS**, as revealed by the computed electronic absorption spectra at the TD-DFT level [41,42]. This second photon absorption at **MS2** triggers electronic transitions to ^1^MLCT states from which non-radiative decays by ISC and IC lead most probably to the population of **^3^MS2**. From this intermediate, only a small adiabatic barrier needs to be overcome on the lowest triplet PES to reach a O-bonded triplet intermediate (denoted **^3^MS1**), in which the Ru–O–N is bent. Another triplet/singlet crossing lying nearby allows for ISC to the O-bonded **MS1** product [67], restoring the collinear conformation of the Cl, Ru, O, and N atoms. This final photoproduct does not absorb in the blue light spectral range, allowing for very high photoconversion yield.

### 2.2. Comparison with the trans-[RuBr(NO)(py)_4_]^2+^ and trans-(Cl,Cl)[RuCl_2_(NO)(tpy)]^+^ Complexes

#### 2.2.1. Thermal Isomerization Pathway

The stationary points characterizing the singlet PES along the thermal isomerization pathway were located for all three complexes. They correspond to the **GS**, **MS2,** and **MS1** minima and the two transition states (**TS_1_** and **TS_2_**) connecting them, as shown in Figure 2. Relative energies are given in Table 1. All three complexes display similar potential energy profiles with small changes (<0.1 eV) in the relative energies of the three isomers. The energy barriers **GS** to **MS2** and **MS2** to **MS1** are also comparable in all the complexes with deviations below 0.15 eV. Overall, these results confirm the thermal stability of the stable N-bonded isomer **GS** for each complex.

#### 2.2.2. Adiabatic Photoisomerization Pathway

A similar work performed for the singlet PES was carried out for the lowest triplet PES, allowing a characterization of the adiabatic photoisomerization pathway for the three complexes. The stationary points found on the lowest triplet PES correspond to the **^3^GS**, **^3^MS2,** and **^3^MS1** minima and the two transition states (**^3^TS_1_** and **^3^TS_2_**) connecting them, as shown in Figure 2. Relative energies for these various structures are given in Table 2. As for the thermal ground-state isomerization pathways, the adiabatic triplet photoisomerization pathways are very similar in the three complexes, all involving the same stationary points and comparable relative energies. From a structural point of view, all the optimized triplet structures are analogous in the three complexes. The electronic nature of the lowest triplet state is very similar as well: it can be described as a ^3^MLCT state of Ru(d)→NO(π*) character in all three complexes. A minor difference is found in complex (**2**), for which the bromide ligand presents slightly higher spin densities than the chloride ligand. As far as the energy profiles are concerned, they are qualitatively similar in the three complexes, involving a large adiabatic potential energy barrier (ca. 1 eV) along the photoisomerization pathway. Thus, in all these systems, the N to O linkage photoisomerization cannot occur adiabatically. Note that the three triplet states **^3^GS**, **^3^MS2,** and **^3^MS1** are lowered in energy (by a few tenths of eV; see Table 2) relative to **GS** in complex (**3**).

#### 2.2.3. Nonadiabatic Photoisomerization Pathway

Because of the large barrier occurring along the adiabatic triplet photoisomerization pathway, funnels for efficient non-radiative decay paths between the lowest triplet excited state and the singlet ground state were located by optimizing minimum energy crossing points (MECP) between these two states. As shown in Figure 2, four MECP were located in complex (**1**). The first crossing (**MECP_1_**) provides a photostabilizing pathway for **GS**, as the main relaxation path from this funnel is expected to regenerate the initial stable N-bonded isomer. The second one (**MECP_2_**) allows the system to undergo a partial nonadiabatic photoisomerization from **GS** to **MS2**. The third crossing (**MECP_3_**) provides a photostabilizing pathway for **MS2,** and the last one (**MECP_4_**) allows the system to evolve to the final O-bonded isonitrosyl photoproduct **MS1**. All these four funnels were also found for complexes (**2**) and (**3**). All these critical crossing points are located at similar relative energies compared to the reference complex (**1**), providing similar radiationless pathways for these two complexes (Table 3). The energy required to reach **MECP_2_** is of particular importance in the nonadiabatic photoisomerization mechanism, as it represents the highest activation energy that the system has to overcome along the photoisomerization pathway. This crossing is located at 0.67 and 0.65 eV above **^3^GS** in complexes (**1**) and (**2**), respectively. However, a slightly higher energy is required at 0.90 eV for complex (**3**).

#### 2.2.4. Absorption Properties

**Figure 3:**
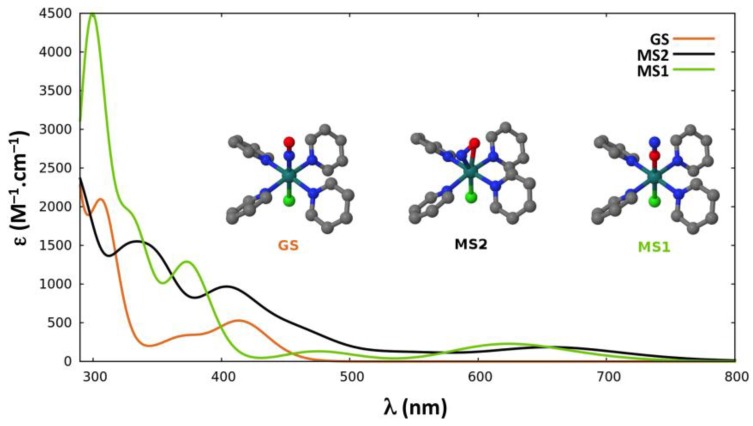
Time-dependent DFT (TD-DFT) absorption spectra of the three linkage isomers (**GS** in orange, **MS2** in black, and **MS1** in green) of *trans*-[RuCl(NO)(py)_4_]^2+^. Broadening model used: Gaussian function with a half-width at half-height of 0.2 eV.

**Figure 4:**
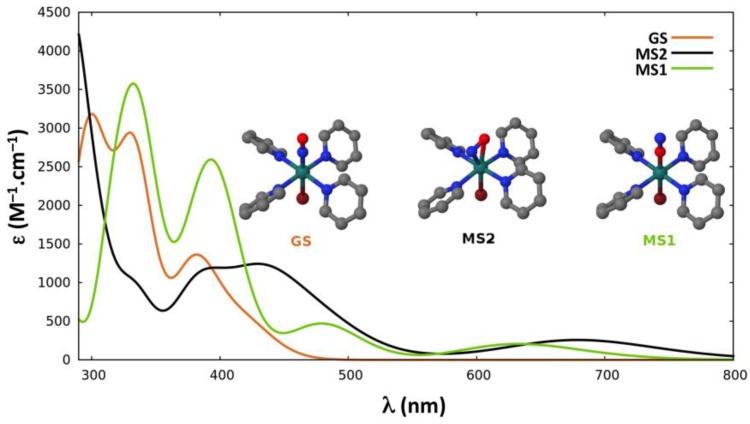
TD-DFT absorption spectra of the three linkage isomers (**GS** in orange, **MS2** in black, and **MS1** in green) of *trans*-[RuBr(NO)(py)_4_]^2+^. Broadening model used: Gaussian function with a half-width at half-height of 0.2 eV.

**Figure 5:**
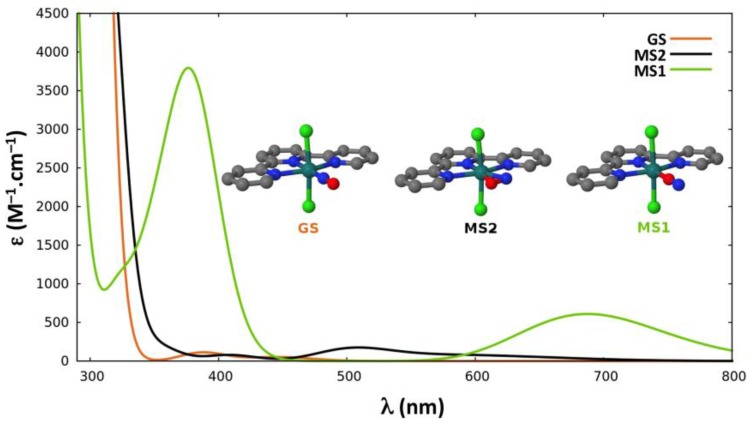
TD-DFT absorption spectra of the three linkage isomers (**GS** in orange, **MS2** in black, and **MS1** in green) of *trans*-(Cl,Cl)[RuCl_2_(NO)(tpy)]^+^. Broadening model used: Gaussian function with a half-width at half-height of 0.2 eV.

In the proposed sequential two-photon photoisomerization mechanism described in Section 2.1, the absorption properties of **GS**, **MS2,** and **MS1** are all of crucial importance. Thus, we report here the TD-DFT absorption spectra computed for each isomer of the three complexes. Figure 3, Figure 4 and Figure 5 display the spectra of **GS**, **MS2,** and **MS1** for complexes (**1**), (**2**) and (**3**), respectively. Further details about the transition wavelengths and oscillators strengths are given in Supporting Information.

In all three complexes, the first significant absorption band of **GS** appears near the experimental irradiation wavelengths (400–500 nm). It is, however, noticeable that the intensity of this band is much lower in complex (**3**). In addition, at the first significant absorption maximum of **GS** around 400 nm, the absorption of **MS1** vanishes in the first complex, whereas it becomes more intense going to complex (**2**) (ε ≈ 2600 M^−1^·cm^−1^) and to complex (**3**) (ε ≈ 3800 M^−1^·cm^−1^). The first absorption band of **MS1** in the red region (650–700 nm) corresponds to a spectral range where the **GS** isomer does not absorb for any of the three complexes. As far as the absorption of **MS2** is concerned, it shows two absorption bands, which strongly overlap with that of the first absorption bands of **GS** at 400–450 nm and of **MS1** at 650–700 nm in the first complex. However, this overlap is very poor in complex (**3**), as **MS2** weakly absorbs around 400 nm and shows no absorption at 700 nm.

## 3. Discussion

Our results show that the photoconversion trend experimentally observed in the family of ruthenium nitrosyl compounds presented in this work can be rationalized making use of the absorption properties of the different isomers (**GS**, **MS2,** and **MS1**) of each complex, rather than changes in the photoisomerization pathway. Indeed, the potential energy profiles for the three complexes are very similar and the slight changes in the relative energies of the critical points along the photoisomerization pathway cannot account for the large variation of photoconversion yields observed. As pointed out in refs. [41,42], and as recalled in Section 2.1, one of the key factors necessary for the photoreactivity of this kind of compounds is the overlap between the absorption bands of the **GS** and **MS2** isomers. This is a requirement for the description of a sequential two-photon mechanism, where, initially, **GS** absorbs one photon and, subsequently, **MS2** absorbs a second one. In the particular case of complex (**1**), which shows the highest photoconversion yield, two remarkable characteristics of the spectra permit the rationalization of the mechanism and experimental yield: a) there is a spectral region near the experimental irradiation wavelengths (400–500 nm) where an important superposition of the absorption bands of **GS** and **MS2** exists; and b) **MS1** does not absorb in that specific region. While the first property is obviously a *sine qua non* condition for a sequential two-photon absorption mechanism, the second property is necessary to achieve high photoconversion yields under continuous irradiation. If the system is progressively transformed from **GS** to **MS1** without **MS1** being consumed, as it does not absorb at the irradiation wavelength, a quantitative conversion of **GS** into **MS1** can be reached.

In the case of complex (**2**), there is also a spectral region (400–500 nm) where an important overlap between the absorption bands of **GS** and **MS2** exists. But in contrast to complex (**1**), **MS1** also presents an absorption band in that same region overlapping those of **GS** and **MS2**. We believe that this is the main reason for lowering by about half the photoconversion yield upon substituting the chloride by the bromide ligand.

Another aspect that can be discussed regards the optimal irradiation wavelength used for photoconversion. During the experimental irradiation times, **MS1** is formed and consumed continuously, and, depending on the chemical rates for the forward and backward isomerizations, different photoconversion yields will be attained once the photostationary state is reached. The fact that the optimal irradiation wavelength found for the complex with the bromide ligand (488–496 nm) [53] is higher with respect to the one found for the complex with the chloride ligand (473–476 nm) [51,52,53] can be explained comparing the different spectra. The optimal irradiation wavelength for that complex should be one where **GS** and **MS2** absorb significantly compared to **MS1**. The important region of the spectra where both **GS** and **MS2** isomers absorb but not **MS1** in complex (**1**) is blue-shifted with respect to the corresponding spectral region in complex (**2**) (i.e., the region where **GS** and **MS2** both absorb and **MS1** shows a weak absorption).

Regarding the low photoconversion yield observed for complex (**3**), it can also be rationalized following the same line of thinking. As shown in Figure 5, in the spectral range of 350–450 nm, **MS1** presents an important absorption band, while the isomers **GS** and **MS2** barely absorb in that region. This is consistent with the very low photoconversion yield observed for this complex. Moreover, it appears that, for this particular complex, there is no wavelength that would trigger an efficient photoisomerization in the 350–500 nm range, as there is no region where both **GS** and **MS2** would absorb and **MS1** would not. In addition, note that for this complex, a slightly higher **^3^GS**→**MS2** barrier (via **MECP_2_**) is encountered.

## 4. Computational Details

Gas-phase geometry optimizations of all the stationary points found on the lowest singlet and triplet PES were carried out using DFT with the Gaussian 09 quantum package [69], with the exception of the transition states in complex (**3**) (see Table 1 and Table 2), for which the Zero Temperature String (ZTS) method [70] was used [42]. The nature of these stationary points (i.e., minima or transition states) was determined by harmonic frequency analyses. These geometry optimizations were performed using the standard hybrid functional B3LYP [71], including Grimme’s dispersion correction [72] (denoted B3LYP-D3), with a double-*ζ* Ahlrichs-type basis set with a set of *p* polarization functions for the hydrogen atoms [73], a triple- *ζ* Ahlrichs-type basis set with one set of *d* polarization functions for all heavy atoms [73], and a Stuttgart relativistic effective small-core potential [74] with its associated basis set including two sets of *f* and one set of *g* polarization functions [75] for ruthenium.

The UV-Vis absorption spectra of **GS**, **MS1,** and **MS2** were computed at the TD-DFT level using the TPSSh functional [76] for complexes (**1**) and (**2**) (Figure 3 and Figure 4), as in ref. [41]. For complex (**3**), the BHandHLYP [77] functional was used (Figure 5) as in ref. [42], along with the TPSSh functional for the sake of comparison (Figure S1 in Supporting Information). The Tamm-Dancoff approximation (TDA) [78] and the same basis sets as described above were used. In these TD-DFT calculations the resolution-of-identity (RI) approximation for hybrid functionals [79] was employed to calculate the Coulomb energy term using the Def2-TZV auxiliary basis set and the exchange term by the so-called ‘chain-of-spheres exchange’ algorithm [80]. All the TD-DFT calculations were performed with the ORCA 3.0.3 quantum package [81].

## 5. Conclusions

We have theoretically investigated the complex N→O linkage photoisomerization pathways along with the absorption properties of all the stable and metastable isomers involved in a series of three ruthenium nitrosyl complexes exhibiting very different photoconversion yields upon continuous blue-light irradiation. Our results unambiguously point towards an increasing absorption of the isonitrosyl photoproduct at the irradiation wavelength used to trigger the N→O linkage photoisomerization to explain the decrease of the photoconversion yield going from *trans*-[RuCl(NO)(py)_4_]^2+^, to *trans*-[RuBr(NO)(py)_4_]^2+^, and to *trans*-(Cl,Cl)[RuCl_2_(NO)(tpy)]^+^.

## Figures and Tables

**Table 1:** Relative energies (eV) for all stationary points on the singlet potential energy surface for each complex.

Relative Energy	*trans*-[RuCl(NO)(py)_4_]^2+^ (1)	*trans*-[RuBr(NO)(py)_4_]^2+^ (2)	*trans*-(Cl,Cl)[RuCl_2_(NO)(tpy)]^+^ (3)
ΔE(**MS2**–**GS**)	1.56	1.51	1.48
ΔE(**MS1**–**GS**)	1.86	1.82	1.91
ΔE(**TS_1_**–**GS**)	2.44	2.30	2.30 ^1^
ΔE(**TS_2_**–**MS2**)	1.57	1.49	1.44 ^1^

^1^ Values obtained with Zero Temperature String (ZTS) calculations [42].

**Table 2:** Relative energies (eV) for all stationary points on the triplet potential energy surface for each complex.

Relative Energy	*trans*-[RuCl(NO)(py)_4_]^2+^ (1)	*trans*-[RuBr(NO)(py)_4_]^2+^ (2)	*trans*-(Cl,Cl)[RuCl_2_(NO)(tpy)]^+^ (3)
ΔE(**^3^GS**–**GS**)	1.41	1.32	0.98
ΔE(**^3^MS2**–**GS**)	2.25	2.19	1.85
ΔE(**^3^MS1**–**GS**)	2.08	1.98	1.70
ΔE(**^3^TS_1_**–**^3^GS**)	1.06	1.05	0.98 ^2^
ΔE(**^3^TS_2_**–**^3^MS2**)	0.25	0.20 ^1^	0.16 ^2^

^1^ Without explicit dispersion. ^2^ Values obtained with Zero Temperature String (ZTS) calculations [42].

**Table 3:** Relative energies (eV) for selected critical points in the studied complexes.

Relative Energy	*trans*-[RuCl(NO)(py)_4_]^2+^ (1)	*trans*-[RuBr(NO)(py)_4_]^2+^ (2)	*trans*-(Cl,Cl)[RuCl_2_(NO)(tpy)]^+^ (3)
ΔE(**MECP_1_**–**^3^GS**)	0.00	0.00	0.01
ΔE(**MECP_2_**–**^3^GS**)	0.67	0.65	0.90
ΔE(**MECP_3_**–**^3^MS2**)	0.03	0.03	0.02
ΔE(**MECP_4_**–**^3^MS1**)	0.17	0.18	0.34

## Supplementary Materials

The following are available online: Table S1: Cartesian coordinates for all the stable and metastable isomers; Table S2: TD-TPSSh absorption spectra of *trans*-[RuCl(NO)(py)_4_]^2+^; Table S3: TD-TPSSh absorption spectra of *trans*-[RuBr(NO)(py)_4_]^2+^; Table S4: TD-BHandHLYP absorption spectra of *trans*-(Cl,Cl)[RuCl_2_(NO)(tpy)]^+^; Figure S1: TD-TPSSh absorption spectra of *trans*-(Cl,Cl)[RuCl_2_(NO)(tpy)]^+^.
