# Supplementary material for: A Theoretical Study of the N to O Linkage Photoisomerization Efficiency in a Series of Ruthenium Mononitrosyl Complexes
Source: Molecules. 2017 Oct 6;22(10):1667. doi: 10.3390/molecules22101667 (PMC6151532; doi:10.3390/molecules22101667)
Supplement: Supplementary file 1 [file molecules-22-01667-s001.pdf]

molecules

MDPI

Supporting Information for

A theoretical study of the N to O linkage photoisomerization efficiency in a series of ruthenium mononitrosyl complexes

Juan Sanz García [1,2,*], Francesco Talotta [1], Fabienne Alary [1], Isabelle M. Dixon [1], Jean-Louis Heully [1], and Martial Boggio-Pasqua [1,*]

[1] Laboratoire de Chimie et Physique Quantiques, IRSAMC, CNRS et Université Toulouse 3, 118 route de Narbonne, 31062 Toulouse, France
[2] Institut de Recherche de Chimie Paris, PSL Research University, CNRS, Chimie ParisTech, 11 Rue Pierre et Marie Curie, F-75005 Paris, France
* Correspondence: juan.sanz-garcia@chimie-paristech.fr; Tel.: +33-144-276-728; martial.boggio@irsamc.ups-tlse.fr; Tel.: +33-561-556-833

Table of Contents.

Table S1. Cartesian coordinates for all the stable and metastable isomers p. S2

Table S2. TD-TPSSh absorption spectra for complex (**1**) p. S11

Table S3. TD-TPSSh absorption spectra for complex (**2**) p. S12

Table S4. TD- BHandHLYP absorption spectra for complex (**3**) p. S13

Figure S1. TD-TPSSh absorption spectra for complex (**3**) p. S14

Table S1. B3LYP-D3 optimized Cartesian coordinates for **GS** of *trans*-$[RuCl(NO)(py)_4]^{2+}$.

Ru	0.000000000000	0.000000000000	0.075373000098
Cl	0.000000000000	0.000000000000	-2.241876998087
N	0.000715000003	2.137759000999	0.008602000003
N	2.137759000999	-0.000715000003	0.008602000003
N	-0.000715000003	-2.137759000999	0.008602000003
N	-2.137759000999	0.000715000003	0.008602000003
N	0.000000000000	0.000000000000	1.820093001914
O	0.000000000000	0.000000000000	2.960970001222
C	-0.805272998832	2.842570998263	0.832481999705
H	-1.447122999683	2.277536001171	1.498677999269
C	-0.833355999367	4.224145002329	0.833475000737
H	-1.498087998432	4.743048002577	1.516766001232
C	-0.008361999988	4.918275002001	-0.044745999978
H	-0.012454000022	6.004203996837	-0.067041000172
C	0.818857999089	4.194434998841	-0.893259002257
H	1.477028999825	4.689415998321	-1.600218998603
C	0.801454001002	2.811346997614	-0.842417000166
H	1.422719998000	2.219019998203	-1.501717000477
C	2.842570998263	0.805272998832	0.832481999705
H	2.277536001171	1.447122999683	1.498677999269
C	4.224145002329	0.833355999367	0.833475000737
H	4.743048002577	1.498087998432	1.516766001232
C	4.918275002001	0.008361999988	-0.044745999978
H	6.004203996837	0.012454000022	-0.067041000172
C	4.194434998841	-0.818857999089	-0.893259002257
H	4.689415998321	-1.477028999825	-1.600218998603
C	2.811346997614	-0.801454001002	-0.842417000166
H	2.219019998203	-1.422719998000	-1.501717000477
C	-0.801454001002	-2.811346997614	-0.842417000166
H	-1.422719998000	-2.219019998203	-1.501717000477
C	-0.818857999089	-4.194434998841	-0.893259002257
H	-1.477028999825	-4.689415998321	-1.600218998603
C	0.008361999988	-4.918275002001	-0.044745999978
H	0.012454000022	-6.004203996837	-0.067041000172
C	0.833355999367	-4.224145002329	0.833475000737
H	1.498087998432	-4.743048002577	1.516766001232
C	0.805272998832	-2.842570998263	0.832481999705
H	1.447122999683	-2.277536001171	1.498677999269
C	-2.811346997614	0.801454001002	-0.842417000166
H	-2.219019998203	1.422719998000	-1.501717000477
C	-4.194434998841	0.818857999089	-0.893259002257
H	-4.689415998321	1.477028999825	-1.600218998603
C	-4.918275002001	-0.008361999988	-0.044745999978
H	-6.004203996837	-0.012454000022	-0.067041000172
C	-4.224145002329	-0.833355999367	0.833475000737
H	-4.743048002577	-1.498087998432	1.516766001232
C	-2.842570998263	-0.805272998832	0.832481999705
H	-2.277536001171	-1.447122999683	1.498677999269

Table S1 (continued). B3LYP-D3 optimized Cartesian coordinates for **MS2** of *trans*-$[RuCl(NO)(py)_4]^{2+}$.

Ru	-0.161797675233	-0.000484407770	0.022594707618
Cl	2.132925867041	-0.103880706629	0.165861360927
N	0.083633431350	1.344873189658	-1.614848799801
N	-0.162393057376	1.692196007102	1.316110093760
N	-0.011115130559	-1.392250851126	1.699055774043
N	-0.132674199670	-1.642858624511	-1.348445084001
N	-1.984203797413	-0.412946041402	0.489462774945
O	-2.260753090415	0.341932163466	-0.369527152746
C	-0.664316061446	1.195378380425	-2.730440934715
H	-1.364140101740	0.368780923531	-2.748848376419
C	-0.554225189227	2.051264722659	-3.809385206617
H	-1.180649821981	1.890519957103	-4.681020799813
C	0.359752989653	3.097686555244	-3.754191609167
H	0.469129068477	3.781791028862	-4.590799848132
C	1.131302553858	3.248545351301	-2.609194408008
H	1.861074496820	4.046795436002	-2.518967497816
C	0.971029434446	2.360521723544	-1.560855419004
H	1.566777526570	2.444057537796	-0.661304274577
C	-1.006171474327	2.718401300438	1.073025093180
H	-1.646045811992	2.637642940404	0.201612343578
C	-1.062005384432	3.837949009890	1.882216693869
H	-1.761751342559	4.632900514786	1.644936762203
C	-0.211455447403	3.922323716492	2.978620121276
H	-0.229549657263	4.793189158319	3.627548033528
C	0.666004833554	2.873460930116	3.223172193433
H	1.354780227173	2.895822259731	4.061701370389
C	0.666199853205	1.777609452481	2.378430682023
H	1.345078142165	0.949562989459	2.535996491123
C	0.727675982304	-2.515114817405	1.594835821961
H	1.247218673855	-2.678067564604	0.660332355842
C	0.845032606771	-3.416117097934	2.637932693388
H	1.453738705447	-4.304515329344	2.503445097704
C	0.189212382599	-3.161553525606	3.835482256167
H	0.269929373851	-3.851798797145	4.670184877359
C	-0.572008988708	-2.004350743764	3.943063649372
H	-1.105249217858	-1.758513579943	4.855781426645
C	-0.652351501202	-1.148748765340	2.859804091673
H	-1.245906764946	-0.244142228387	2.918795828141
C	0.784532149054	-1.691974633538	-2.338848399727
H	1.496758271756	-0.878450568479	-2.386344460658
C	0.836556831110	-2.735036880111	-3.246720169992
H	1.597790355583	-2.725304341512	-4.020323905759
C	-0.080551561815	-3.772902093129	-3.146382490335
H	-0.060091956959	-4.602576852577	-3.847066601149
C	-1.022705489124	-3.729495787241	-2.125180705883
H	-1.758343491951	-4.517432055830	-1.998208211299
C	-1.017220996704	-2.660734944204	-1.249071238130
H	-1.731109554554	-2.618909001647	-0.433494002146

Table S1 (continued). B3LYP-D3 optimized Cartesian coordinates for **MS1** of *trans*-[RuCl(NO)(py)$_4$]$^{2+}$.

Ru	0.000000000000	0.000000000000	0.000209000000
Cl	0.000000000000	0.000000000000	-2.277904000000
N	-0.000060000000	2.128222000000	-0.017851000000
N	2.128222000000	0.000060000000	-0.017851000000
N	0.000060000000	-2.128222000000	-0.017851000000
N	-2.128222000000	-0.000060000000	-0.017851000000
O	0.000000000000	0.000000000000	1.853867000000
N	0.000000000000	0.000000000000	2.990883000000
C	-0.799437000000	2.811765000000	0.829567000000
H	-1.445463000000	2.229088000000	1.477124000000
C	-0.821844000000	4.193094000000	0.875420000000
H	-1.481827000000	4.693644000000	1.576750000000
C	-0.000061000000	4.910623000000	0.013217000000
H	-0.000874000000	5.996718000000	0.023445000000
C	0.818178000000	4.209959000000	-0.863487000000
H	1.472536000000	4.724674000000	-1.559834000000
C	0.796798000000	2.826084000000	-0.853375000000
H	1.414134000000	2.250874000000	-1.531766000000
C	2.811765000000	0.799437000000	0.829567000000
H	2.229088000000	1.445463000000	1.477124000000
C	4.193094000000	0.821844000000	0.875420000000
H	4.693644000000	1.481827000000	1.576750000000
C	4.910623000000	0.000061000000	0.013217000000
H	5.996718000000	0.000874000000	0.023445000000
C	4.209959000000	-0.818178000000	-0.863487000000
H	4.724674000000	-1.472536000000	-1.559834000000
C	2.826084000000	-0.796798000000	-0.853375000000
H	2.250874000000	-1.414134000000	-1.531766000000
C	-0.796798000000	-2.826084000000	-0.853375000000
H	-1.414134000000	-2.250874000000	-1.531766000000
C	-0.818178000000	-4.209959000000	-0.863487000000
H	-1.472536000000	-4.724674000000	-1.559834000000
C	0.000061000000	-4.910623000000	0.013217000000
H	0.000874000000	-5.996718000000	0.023445000000
C	0.821844000000	-4.193094000000	0.875420000000
H	1.481827000000	-4.693644000000	1.576750000000
C	0.799437000000	-2.811765000000	0.829567000000
H	1.445463000000	-2.229088000000	1.477124000000
C	-2.826084000000	0.796798000000	-0.853375000000
H	-2.250874000000	1.414134000000	-1.531766000000
C	-4.209959000000	0.818178000000	-0.863487000000
H	-4.724674000000	1.472536000000	-1.559834000000
C	-4.910623000000	-0.000061000000	0.013217000000
H	-5.996718000000	-0.000874000000	0.023445000000
C	-4.193094000000	-0.821844000000	0.875420000000
H	-4.693644000000	-1.481827000000	1.576750000000
C	-2.811765000000	-0.799437000000	0.829567000000
H	-2.229088000000	-1.445463000000	1.477124000000

Table S1 (continued). B3LYP-D3 optimized Cartesian coordinates for **GS** of *trans*-$[RuBr(NO)(py)_4]^{2+}$.

Ru	0.000000004426	0.000000014094	0.038959612966
Br	0.000000028364	0.000000031739	-2.429146995895
N	0.004245119967	2.142946638663	-0.012134657801
N	2.142946630478	-0.004245122531	-0.012134636352
N	-0.004245123938	-2.142946612543	-0.012134657230
N	-2.142946620709	0.004245121386	-0.012134678705
N	-0.000000014134	0.000000016844	1.787769678539
O	-0.000000010727	0.000000019447	2.928669897692
C	-0.818198272081	2.835447185809	0.806150661726
H	-1.472995757073	2.260542902645	1.450711943561
C	-0.847369462180	4.216935922607	0.828975938082
H	-1.526684186466	4.724133143292	1.506688652050
C	-0.004434754835	4.925743803777	-0.019735839177
H	-0.009037644810	6.011880464991	-0.024950857626
C	0.842316549129	4.215525756242	-0.860378102999
H	1.516410063235	4.721586806243	-1.544119985287
C	0.824114806836	2.831893227084	-0.832421130251
H	1.462429564816	2.251889633877	-1.486105975926
C	2.835447175659	0.818198320276	0.806150634085
H	2.260542891012	1.472995858273	1.450711860771
C	4.216935912391	0.847369498498	0.828975926400
H	4.724133130889	1.526684267126	1.506688597573
C	4.925743796308	0.004434721908	-0.019735779714
H	6.011880457647	0.009037599833	-0.024950783464
C	4.215525751207	-0.842316637689	-0.860377989429
H	4.721586803437	-1.516410209470	-1.544119813200
C	2.831893221769	-0.824114879019	-0.832421036941
H	2.251889631291	-1.462429683179	-1.486105839721
C	-0.824114958279	-2.831893203953	-0.832420980164
H	-1.462429836089	-2.251889613835	-1.486105711175
C	-0.842316709056	-4.215525733388	-0.860377941455
H	-1.516410347496	-4.721586785377	-1.544119699697
C	0.004434742063	-4.925743778722	-0.019735824177
H	0.009037628127	-6.011880439987	-0.024950837227
C	0.847369599773	-4.216935894931	0.828975801413
H	1.526684442662	-4.724133113393	1.506688398178
C	0.818198407631	-2.835447158289	0.806150523632
H	1.472996009342	-2.260542874018	1.450711686030
C	-2.831893209207	0.824114886000	-0.832421073642
H	-2.251889616323	1.462429717460	-1.486105847681
C	-4.215525738360	0.842316620494	-0.860378055159
H	-4.721586788093	1.516410201168	-1.544119872028
C	-4.925743786161	-0.004434774787	-0.019735883596
H	-6.011880447300	-0.009037672819	-0.024950911311
C	-4.216935905158	-0.847369563163	0.828975813263
H	-4.724133125845	-1.526684361554	1.506688452953
C	-2.835447168450	-0.818198359249	0.806150551397
H	-2.260542885687	-1.472995907880	1.450711769040

Table S1 (continued). B3LYP-D3 optimized Cartesian coordinates for **MS2** of *trans*-[RuBr(NO)(py)$_4$]$^{2+}$.

Ru	-0.123933218654	-0.001444011648	0.025625669944
Br	2.320412754017	-0.117331474807	0.186088605588
N	0.106850009729	1.351023043121	-1.613402493730
N	-0.141647992532	1.697068774009	1.319945458121
N	0.011881334202	-1.404072002161	1.702596401069
N	-0.110322444526	-1.644373364382	-1.350306519809
N	-1.950043480512	-0.410425150617	0.496282918179
O	-2.243727640405	0.337517900006	-0.360991281908
C	-0.639461426731	1.185161040261	-2.728073366705
H	-1.321513820086	0.344008534225	-2.744820210116
C	-0.551894758527	2.042620893111	-3.807926136870
H	-1.176657252762	1.866599936744	-4.677796383238
C	0.336683252996	3.110658733484	-3.755420863684
H	0.428528977116	3.796466374982	-4.592737121522
C	1.104526714010	3.281467107940	-2.610767544163
H	1.813870627855	4.097994937379	-2.521047539735
C	0.967178658542	2.390375300280	-1.561948253408
H	1.560489325510	2.492060381596	-0.662813781748
C	-0.981102028879	2.721164482864	1.052484756336
H	-1.599390144214	2.635955166052	0.166283483845
C	-1.060525669144	3.844099820383	1.855127041319
H	-1.756096072478	4.635954972980	1.596217913797
C	-0.239214728089	3.935630285628	2.972778310220
H	-0.275865532224	4.809224340735	3.617225669735
C	0.632397913325	2.888774410453	3.244941399095
H	1.298231637748	2.914974630735	4.101706658182
C	0.656620592444	1.789628616172	2.405014944084
H	1.331078540257	0.963277901609	2.587609448425
C	0.710451905708	-2.552603564258	1.595328519054
H	1.219698250099	-2.733857091894	0.658612443669
C	0.801535574740	-3.458706805435	2.636356803655
H	1.379108981443	-4.367042703899	2.497689840559
C	0.160735745746	-3.183594550174	3.837550949823
H	0.222211459663	-3.876887944231	4.671348250243
C	-0.561600675558	-2.002364602860	3.948394280651
H	-1.084184667545	-1.740190879616	4.862723772098
C	-0.617992056919	-1.143611381777	2.865778590087
H	-1.185033998854	-0.222614850400	2.927776711996
C	0.777748045450	-1.690885660996	-2.367389140735
H	1.485088024052	-0.874830686063	-2.436676405995
C	0.807178918826	-2.732637913839	-3.277649565364
H	1.546266144855	-2.719481070813	-4.072397109381
C	-0.103562886008	-3.773362654636	-3.152913198260
H	-0.100455733404	-4.602324713161	-3.854721922465
C	-1.017311568892	-3.732557044602	-2.106239033118
H	-1.748161796116	-4.521624674789	-1.960125949355
C	-0.989857801266	-2.664624002638	-1.229485001621
H	-1.681869004222	-2.625213775424	-0.395307018588

Table S1 (continued). B3LYP-D3 optimized Cartesian coordinates for **MS1** of *trans*-$[RuBr(NO)(py)_4]^{2+}$.

Ru	-0.000000007623	-0.000000009470	-0.040424835210
Br	-0.000000019456	0.000000002647	-2.462732789494
N	0.002946494564	2.132844414744	-0.040589861327
N	2.132844416294	-0.002946492843	-0.040589879434
N	-0.002946492788	-2.132844433211	-0.040589886766
N	-2.132844431649	0.002946494504	-0.040589868629
O	-0.000000000431	-0.000000018617	1.822214035514
N	-0.000000018592	-0.000000030551	2.959436535501
C	-0.811571229550	2.803524466445	0.802985975859
H	-1.469587236084	2.211113927028	1.428954996777
C	-0.834756511685	4.184002050733	0.872420762651
H	-1.508244005251	4.672239088715	1.569581408638
C	0.003855764779	4.916295966375	0.039642192393
H	0.002776884055	6.002052441586	0.068315996861
C	0.840317833792	4.229955673421	-0.831106064376
H	1.509894531885	4.755779135713	-1.504314878382
C	0.817929366952	2.846240808120	-0.845396492111
H	1.451849892465	2.284028053840	-1.519143249045
C	2.803524466443	0.811571278246	0.802985913533
H	2.211113925269	1.469587309425	1.428954907007
C	4.184002051060	0.834756578401	0.872420687651
H	4.672239087743	1.508244110419	1.569581297400
C	4.916295968593	-0.003855729385	0.039642150609
H	6.002052444037	-0.002776835657	0.068315945797
C	4.229955677217	-0.840317845905	-0.831106061784
H	4.755779140998	-1.509894569630	-1.504314849143
C	2.846240811547	-0.817929394231	-0.845396479301
H	2.284028058524	-1.451849957193	-1.519143202000
C	-0.817929334603	-2.846240823646	-0.845396551104
H	-1.451849845413	-2.284028066363	-1.519143319442
C	-0.840317787759	-4.229955689325	-0.831106143212
H	-1.509894461287	-4.755779149166	-1.504314983574
C	-0.003855735424	-4.916295985735	0.039642126908
H	-0.002776843983	-6.002052461343	0.068315915932
C	0.834756512256	-4.184002073143	0.872420728873
H	1.508243993176	-4.672239114007	1.569581385048
C	0.811571218188	-2.803524488098	0.802985960414
H	1.469587203265	-2.211113950355	1.428955005532
C	-2.846240820207	0.817929307270	-0.845396563928
H	-2.284028061665	1.451849780571	-1.519143366556
C	-4.229955685516	0.840317775605	-0.831106145796
H	-4.755779143877	1.509894423456	-1.504315012844
C	-4.916295983494	0.003855770843	0.039642168770
H	-6.002052458868	0.002776892418	0.068315967090
C	-4.184002072790	-0.834756445474	0.872420803993
H	-4.672239114952	-1.508243887897	1.569581496452
C	-2.803524488074	-0.811571169443	0.802986022831
H	-2.211113952080	-1.469587129837	1.428955095425

Table S1 (continued). B3LYP-D3 optimized Cartesian coordinates for **GS** of *trans*-(Cl,Cl)[$RuCl_2$(NO)(tpy)]$^+$.

Ru	3.359060564218	0.053046264782	-0.061801673164
Cl	3.126959144576	-0.093564109673	-2.429503212980
Cl	3.163259296268	0.173226719527	2.310787511369
N	5.096082712590	0.159784820963	-0.079884868709
N	1.330149798790	-0.072049755274	-0.039040009727
N	2.805868860327	2.067770292163	-0.171438743501
N	3.060101111288	-2.014181321133	0.056617448801
O	6.233601996745	0.229442623279	-0.090645845467
C	0.626372706245	1.067844058972	-0.097970234280
C	1.460265903665	2.280026505233	-0.173068663941
H	-2.457071383857	-0.305640861633	0.002887539160
C	3.180980835353	4.416926607611	-0.308248926867
H	3.896433533899	5.230664489072	-0.360231518407
C	0.773225096040	-1.289686377267	0.033933535162
C	-0.612525974529	-1.400218221001	0.050824531070
H	-1.094698252236	-2.368555787968	0.109201994411
C	3.723956700937	-4.299436421429	0.179011777088
H	4.534956531326	-5.019079917799	0.212742979169
C	1.751161270029	-2.390160672040	0.088115940582
C	-1.373595638311	-0.238810913977	-0.009030234247
C	0.947056638050	3.567153137585	-0.242395855863
H	-0.124976019776	3.726491414187	-0.243223962921
C	-0.762495165191	1.007227893734	-0.084062774887
H	-1.361179505220	1.908886281033	-0.130546948588
C	3.639569741860	3.110233025142	-0.237424399744
H	4.700413512283	2.890973887647	-0.233426459849
C	4.016828948404	-2.946328278742	0.100962690781
H	5.042375577872	-2.598525224817	0.073069764446
C	1.401669452451	-3.730570205795	0.165812601320
H	0.357624220776	-4.020423830268	0.190262407980
C	2.396821311679	-4.697634147389	0.211940356959
H	2.134824884239	-5.749310874253	0.272842854407
C	1.814570719298	4.649019761083	-0.310762327832
H	1.424037869914	5.660395138450	-0.365362271731

Table S1 (continued). B3LYP-D3 optimized Cartesian coordinates for **MS2** of *trans*-(Cl,Cl)[$RuCl_2(NO)(tpy)$]$^+$.

Ru	3.360115543841	-0.041933915395	-0.042159884162
Cl	3.275506529709	-0.293434194591	-2.408572691650
Cl	3.274304232110	-0.026544626635	2.338612942289
N	5.110072428252	0.726657323169	-0.080438316918
N	1.352825529905	-0.085588403504	-0.035227107698
N	2.798717259281	2.034500176315	-0.164292075612
N	3.010958469179	-2.092699444995	0.073300827605
O	5.451025589757	-0.398051600667	-0.020746873108
C	0.639563505578	1.050036845477	-0.096527319580
C	1.459619156567	2.256051760502	-0.171767544863
H	-2.462872328494	-0.290995332082	-0.000783781783
C	3.164039905440	4.389457847636	-0.312117017479
H	3.881789852767	5.201073883223	-0.366445080685
C	0.764800410364	-1.300278570213	0.038328858782
C	-0.620128883995	-1.393102571255	0.051645494035
H	-1.103688109468	-2.360516966131	0.110420594138
C	3.617011933654	-4.388595865048	0.198452554015
H	4.409204529039	-5.128612982473	0.235808089800
C	1.696864160528	-2.430685864780	0.098230678549
C	-1.379114537067	-0.232182929758	-0.010751694201
C	0.937532085843	3.541153667504	-0.248078403130
H	-0.135182732320	3.693746061181	-0.252551113730
C	-0.751065138852	0.999934103582	-0.085528711429
H	-1.333631589609	1.911627697584	-0.134456962820
C	3.624982007648	3.084376971581	-0.233983660072
H	4.686905097839	2.881200581800	-0.227401773722
C	3.947637873276	-3.044701937583	0.121712981583
H	4.980250690996	-2.723251200330	0.098264597621
C	1.309783678360	-3.761115637325	0.174494903280
H	0.258985513663	-4.024480039056	0.194023051426
C	2.279630899644	-4.752434571335	0.225416902682
H	1.990513454948	-5.797072468809	0.285294826302
C	1.798382039043	4.624926425579	-0.319073113176
H	1.406760942577	5.635546776833	-0.379349176292

Table S1 (continued). B3LYP-D3 optimized Cartesian coordinates for **MS1** of *trans*-(Cl,Cl)[$RuCl_2(NO)(tpy)]^+$.

Ru	3.307865993315	0.049511943364	-0.064859082418
Cl	3.172140632048	-0.093611734044	-2.428847964265
Cl	3.221243495436	0.178676056435	2.302406378757
N	1.329275772945	-0.071973443446	-0.037193032940
N	2.794824538949	2.067011833862	-0.176314346314
N	3.048679238521	-2.015424593831	0.056408512158
O	5.153682825118	0.163040960248	-0.088127142489
N	6.284362107555	0.232796915112	-0.099667266675
C	0.621496398618	1.070255841742	-0.095841506095
C	1.451119646182	2.283892690344	-0.174755363232
H	-2.462246105271	-0.304834719814	0.014661548242
C	3.177591146670	4.414097373981	-0.317425561473
H	3.894799205211	5.226086515927	-0.372541467382
C	0.768414896459	-1.292093384507	0.039035156939
C	-0.616987497814	-1.398811223315	0.059355488822
H	-1.097861555538	-2.367669110598	0.120194675157
C	3.719459562231	-4.297986754222	0.179858790196
H	4.531854546123	-5.016126543234	0.212136346625
C	1.742106898854	-2.395027829528	0.092322543332
C	-1.378848216072	-0.238291522668	-0.000029735936
C	0.939772758454	3.571909976719	-0.244382422994
H	-0.131704907133	3.735320400135	-0.242621379459
C	-0.766592788859	1.006621194576	-0.078149637994
H	-1.363885057995	1.909245151899	-0.124364932098
C	3.630898573648	3.105126753847	-0.245992458548
H	4.691723091779	2.881412101654	-0.244703414193
C	4.007077087507	-2.943463324205	0.098976932469
H	5.032123743816	-2.591783091726	0.067220459282
C	1.394296588460	-3.735860685683	0.172975381342
H	0.351227223507	-4.029100749724	0.201013466421
C	2.393070847833	-4.699639970660	0.217443656037
H	2.134613067843	-5.752032583364	0.280660370230
C	1.811509993698	4.650471116743	-0.316497214313
H	1.424583247903	5.663190437979	-0.371410777186

Table S2. TD-TPSSh absorption spectra and selected states of the **GS** (orange), **MS1** (green), and **MS2** (black) isomers of the *trans*-$[RuCl(NO)(py)_4]^{2+}$ complex. The nature of the main transitions involved is indicated (+ and – denote a bonding and an antibonding character, respectively, between the fragments in parentheses before the sign).

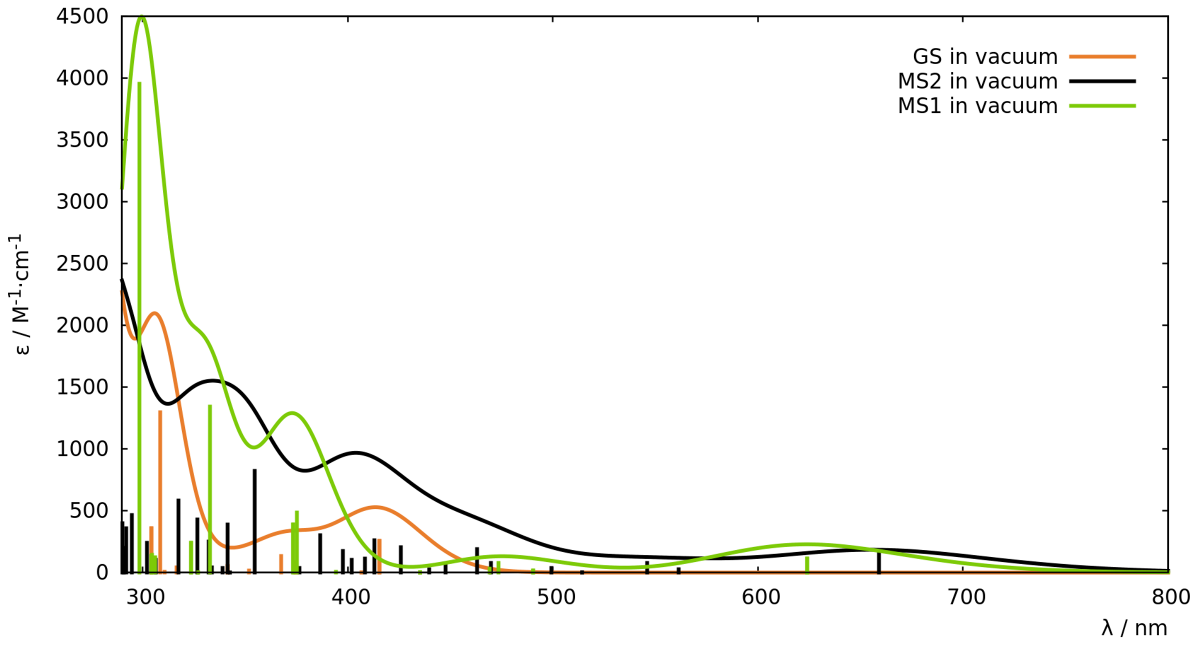

	State	Nature	Wavelength / nm	f_{osc}
**GS**	S_1 & S_2	Ru(d) → (Ru(d)NO(π*))-	416	0.0032
	S_6 & S_7	py(π) → (Ru(d)NO(π*))-	375	0.0010
**MS2**	S_1	Cl(p)Ru(d) → Ru(d)NO(π*)	659	0.0023
	S_6	py(π)Ru(d)NO(π*) → Ru(d)NO(π*)	470	0.0010
	S_7	py(π)Ru(d)NO(π*) → Ru(d)NO(π*)<br>Cl(p)(Ru(d)NO(π*))+ → Cl(p)(Ru(d)NO(π*))-	463	0.0024
	S_{10}	py(π) → Cl(p)Ru(d)NO(π*)	426	0.0026
	S_{11}	py(π)Ru(d)NO(π*) → Ru(d)NO(π*)	413	0.0033
	S_{12}	py(π) → Cl(p)Ru(d)NO(π*)	408	0.0015
	S_{13}	Ru(d)py(π) → Cl(p)Ru(d)NO(π*)	402	0.0013
	S_{14}	Cl(p)Ru(d)py(π) → Ru(d)NO(π*)	398	0.0022
	S_{15}	py(π) → Ru(d)NO(π*)	387	0.0039
**MS1**	S_2 & S_3	Ru(d) → (Ru(d)ON(π*))-	624	0.0015
	S_{17}	Cl(p)py(π) → (Ru(d)ON(π*))-	375	0.0062
	S_{18} & S_{19}	py(π) → (Ru(d)ON(π*))-	373	0.0049

Table S3. TD-TPSSh absorption spectra and selected states of the **GS** (orange), **MS1** (green), and **MS2** (black) isomers of the *trans*-[RuBr(NO)(py)$_4$]$^{2+}$ complex. The nature of the main transitions involved is indicated (+ and – denote a bonding and an antibonding character, respectively, between the fragments in parentheses before the sign).

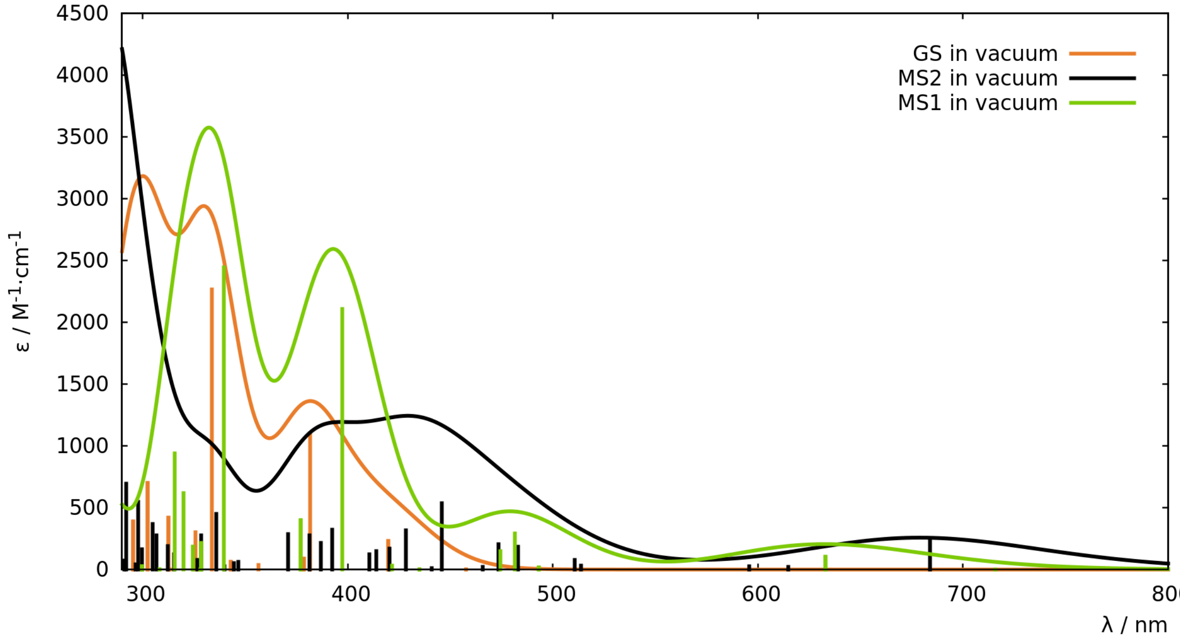

	State	Nature	Wavelength / nm	f_{osc}
**GS**	S$_4$ & S$_5$	Ru(d) → (Ru(d)NO(π*))-	420	0.0029
	S$_6$	(2x) py(π) → (Ru(d)NO(π*))-	382	0.0141
	S$_7$ & S$_8$	py(π) → (Ru(d)NO(π*))-	379	0.0011
**MS2**	S$_1$	Ru(d) → Ru(d)NO(π*)	684	0.0031
	S$_5$	Ru(d)py(π) → Ru(d)NO(π*)<br>Br(p)(Ru(d)NO(π*))+ → Br(p)(Ru(d)NO(π*))-	511	0.0010
	S$_6$	Ru(d)py(π) → Ru(d)NO(π*)<br>py(π)Br(p)Ru(d)NO(π*) → Br(p)Ru(d)NO(π*)	483	0.0024
	S$_7$	Ru(d)py(π) → Ru(d)NO(π*)<br>py(π)Ru(d)NO(π*) → Br(p)Ru(d)NO(π*)	473	0.0026
	S$_9$	Ru(d)py(π) → Br(p)Ru(d)NO(π*)<br>py(π)Ru(d)NO(π*) → Ru(d)NO(π*)	446	0.0069
	S$_{11}$	py(π) → Br(p)Ru(d)NO(π*)	428	0.0040
	S$_{12}$	Ru(d)py(π) → Br(p)Ru(d)NO(π*)	421	0.0021
	S$_{13}$	py(π)Ru(d)NO(π*) → Ru(d)NO(π*)	414	0.0019
	S$_{14}$	py(π)Ru(d)NO(π*) → Ru(d)NO(π*)	411	0.0016
**MS1**	S$_4$ & S$_5$	Ru(d) → (Ru(d)ON(π*))-	633	0.0013
	S$_8$	(2x) Ru(d)py(π) → (Ru(d)ON(π*))-	482	0.0037
	S$_9$	(2x) Ru(d)py(π) → (Ru(d)ON(π*))-	474	0.0019
	S$_{17}$	(2x) py(π) → (Ru(d)ON(π*))-	397	0.0269

Table S4. TD-BHandHLYP absorption spectra and selected states of the **GS** (orange), **MS1** (green), and **MS2** (black) isomers of the *trans*-(Cl,Cl)[RuCl$_2$(NO)(tpy)]$^+$ complex. The nature of the main transitions involved is indicated (+ and – denote a bonding and an antibonding character, respectively, between the fragments in parentheses before the sign).

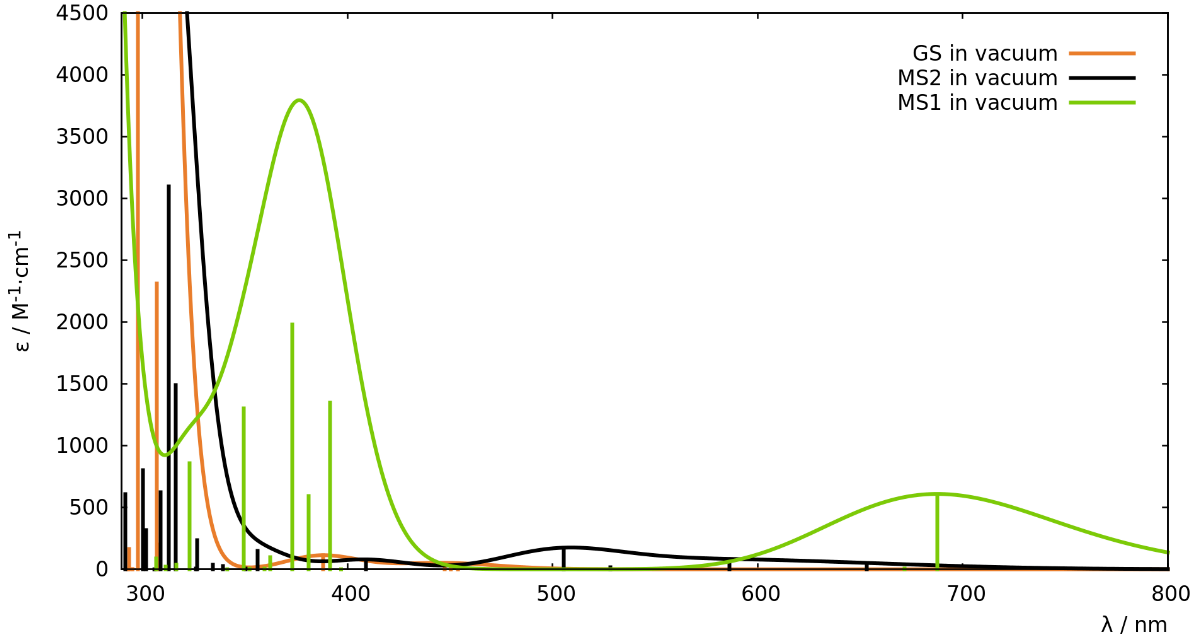

	State	Nature	Wavelength / nm	f_{osc}
**GS**	S$_5$	(Cl$_2$(p)(Ru(d)NO(π*))+)- → (Ru(d)NO(π*))-	388	0.0014
**MS2**	S$_4$	(Cl$_2$(p)(Ru(d))- NO(π*) → Ru(d)NO(π*)	505	0.0020
	S$_6$	((Ru(d)NO(π*))+ → Ru(d)NO(π*)	409	0.0010
	S$_7$	Cl$_2$(p) → (Ru(d)NO(π*))-<br>(Cl$_2$(p)(Ru(d))- NO(π*) → (Ru(d)tpy(σ))-	356	0.0019
**MS1**	S$_3$	(Cl$_2$(p)(Ru(d)NO(π*))+)- → (Ru(d)NO(π*))-<br>((Ru(d)NO(π*))+ → (Ru(d)NO(π*))-	688	0.0077
	S$_8$	Cl$_2$(p) → (Ru(d)NO(π*))-	392	0.0172
	S$_9$	Cl$_2$(p) → (Ru(d)NO(π*))-	381	0.0076
	S$_{10}$	(Ru(d)tpy(π))- → (Ru(d)NO(π*))-	373	0.0253
	S$_{11}$	Cl$_2$(p) → (Ru(d)NO(π*))-<br>Cl$_2$(p)Ru(d) → (Ru(d)NO(π*))-	362	0.0013

Figure S1. TD-TPSSh absorption spectra of the **GS** (orange), **MS1** (green), and **MS2** (black) isomers of the *trans*-(Cl,Cl)[$RuCl_2$(NO)(tpy)]$^+$ complex.

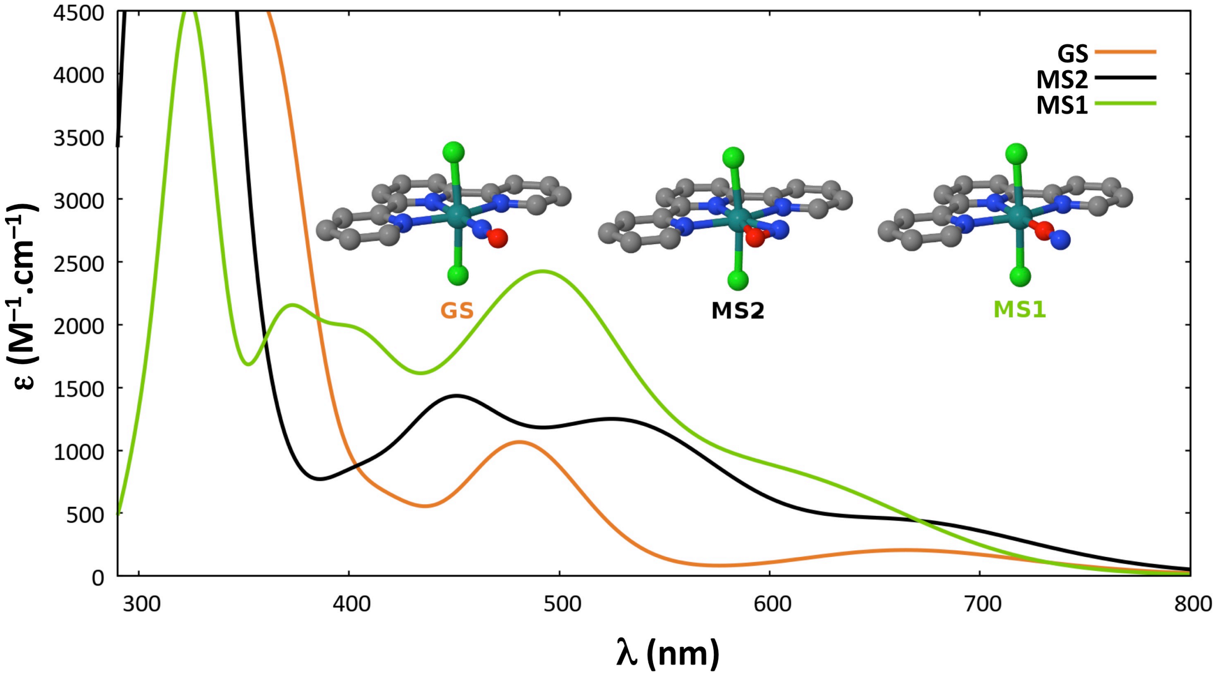

Note that overall the spectra are substantially red-shifted and absorption features are more intense compared to the TD-BHandHLYP results shown in Table S4. In particular, it can be noted that the absorption of **GS** in the blue light region is more intense than that observed with the BHandHLYP functional. Note also that the overlap between the absorption bands of **MS2** with those of **GS** and **MS1** is stronger than the overlap obtained with BHandHLYP. However, upon blue-light irradiation, **MS1** absorbs more significantly than **GS** and **MS2**, which is consistent with the results obtained with BHandHLYP and with the very low photoconversion yield observed experimentally for this complex.
